# Supplementary material for: Quantum-enhanced metrology for multiple phase estimation with noise
Source: Sci Rep. 2014 Aug 4;4:5933. doi: 10.1038/srep05933 (PMC4123202; doi:10.1038/srep05933)
Supplement: Supplementary Information — Supplementary Material: Quantum-enhanced metrology for multiple phase estimation with noise [file srep05933-s1.pdf]

Supplementary Material: Quantum-enhanced metrology for multiple phase estimation with noise

Jie-Dong Yue,[1] Yu-Ran Zhang,[1] and Heng Fan[1,*]
[1] *Beijing National Laboratory for Condensed Matter Physics, Institute of Physics, Chinese Academy of Sciences, Beijing 100190, China*

PACS numbers: 42.50.St, 42.50.Ex, 03.65.Ta, 42.30.-d

A BRIEF REVIEW OF THE QFI APPROACH TO QUANTUM METROLOGY

The quantum Cramér-Rao (QCR) inequality provides a fundamental lower bound $I_Q(\boldsymbol{\theta})$ for the total uncertainty of the multiple phase estimation if the estimator is unbiased [1, 2]

$$Cov(\boldsymbol{\theta}) \geq (MI_Q(\boldsymbol{\theta}))^{-1}, \tag{S1}$$

where the inequality means that $Cov(\boldsymbol{\theta}) - (MI_Q(\boldsymbol{\theta}))^{-1}$ is nonnegative and M stands for classical repetitions. $I_Q(\boldsymbol{\theta})$ is the quantum Fisher information (QFI) matrix, which is defined as

$$[I_Q(\boldsymbol{\theta})]_{ij} = Tr\left[\rho(\boldsymbol{\theta})\frac{L_iL_j + L_jL_i}{2}\right]. \tag{S2}$$

L_i is the symmetric logarithmic derivative (SLD) operator, defined through the equation

$$\frac{L_i\rho(\boldsymbol{\theta}) + \rho(\boldsymbol{\theta})L_i}{2} = \frac{\partial\rho(\boldsymbol{\theta})}{\partial\theta_i}. \tag{S3}$$

On the other hand, we have the classical Cramér-Rao (CR) inequality $Cov(\boldsymbol{\theta}) \geq I(\boldsymbol{\theta})^{-1}$, where $I(\boldsymbol{\theta})$ is the Fisher information (FI) matrix, given by [1]

$$[I(\boldsymbol{\theta})]_{ij} = E\left[\frac{\partial \ln p(x|\boldsymbol{\theta})}{\partial\theta_i}\frac{\partial \ln p(x|\boldsymbol{\theta})}{\partial\theta_j}\right]. \tag{S4}$$

$E[...]$ stands for expectation, and $p(x|\boldsymbol{\theta})$ is the probability of getting result x when the phase is $\boldsymbol{\theta}$. In quantum theory, the general positive operator-valued measure (POVM) formalism states that measurement can be described by a set of positive operators $\{\hat{M}_x\}$, which satisfies $\sum_x \hat{M}_x = \mathbb{I}$ [3]. We then have $p(x) = Tr(\hat{M}_x\rho(\boldsymbol{\theta}))$ and we shall also write $I(\boldsymbol{\theta})$ explicitly as $I(\boldsymbol{\theta}, \hat{M})$.

In general, the relation $I_Q(\boldsymbol{\theta}) \geq \max_{\{\hat{M}\}} I(\boldsymbol{\theta}, \hat{M})$ holds [4]. For the case of single phase estimation, the QCR bound can be achieved locally, that is, for a particular θ, a measurement $\hat{M}$ exists to make $I_Q(\theta) = I(\theta, \hat{M})$. However, when the number of phases is greater than 1, the achievability is no longer guaranteed. A conjecture about one sufficient condition for the achievability of the QCR bound is [5][9]

$$Tr\left[\rho(\boldsymbol{\theta})[L_i, L_j]\right] = 0 \tag{S5}$$

for each i and j. This conjecture is reasonable, since we have known that if $[L_i, L_j] = 0$ for all i and j, then the QFI bound is achievable, and $Tr[\rho(\boldsymbol{\theta})[L_i, L_j]] = 0$ is a weaker condition which doesn't object the stronger one. Taken into consideration that both QFI and FI matrix are symmetric and positive, we also have $I_Q(\boldsymbol{\theta})^{-1} \leq \min_{\hat{M}} I(\boldsymbol{\theta}, \hat{M})^{-1}$ [6].

DERIVATION OF $C_Q(\boldsymbol{\theta}, \hat{\Pi}_l)$

For unitary evolution, $|\psi(\boldsymbol{\theta})\rangle = U(\boldsymbol{\theta})|\psi_0\rangle$ and $\rho(\boldsymbol{\theta}) = |\psi(\boldsymbol{\theta})\rangle\langle\psi(\boldsymbol{\theta})|$. Since $\rho(\boldsymbol{\theta}) = \rho(\boldsymbol{\theta})^2$, we have

$$\frac{\partial\rho(\boldsymbol{\theta})}{\partial\theta_i} = \frac{\partial\rho(\boldsymbol{\theta})^2}{\partial\theta_i} = \rho(\boldsymbol{\theta})\frac{\partial\rho(\boldsymbol{\theta})}{\partial\theta_i} + \frac{\partial\rho(\boldsymbol{\theta})}{\partial\theta_i}\rho(\boldsymbol{\theta}). \tag{S6}$$

Compare with the definition of L_i:

$$\frac{\partial\rho(\boldsymbol{\theta})}{\partial\theta_i}=\frac{L_i\rho(\boldsymbol{\theta})+\rho(\boldsymbol{\theta})L_i}{2}, \tag{S7}$$

we get

$$L_i=2\frac{\partial\rho(\boldsymbol{\theta})}{\partial\theta_i}=2\left(|\frac{\partial\psi(\boldsymbol{\theta})}{\partial\theta_i}\rangle\langle\psi(\boldsymbol{\theta})|+|\psi(\boldsymbol{\theta})\rangle\langle\frac{\partial\psi(\boldsymbol{\theta})}{\partial\theta_i}|\right). \tag{S8}$$

Similarly we have

$$L_j=2\left(|\frac{\partial\psi(\boldsymbol{\theta})}{\partial\theta_j}\rangle\langle\psi(\boldsymbol{\theta})|+|\psi(\boldsymbol{\theta})\rangle\langle\frac{\partial\psi(\boldsymbol{\theta})}{\partial\theta_j}|\right). \tag{S9}$$

Now the QFI matrix can be written as

$$\begin{aligned}
I_Q(\boldsymbol{\theta})_{ij} &= Tr[\rho(\boldsymbol{\theta})\frac{L_iL_j+L_jL_i}{2}] \\
&= \langle\psi(\boldsymbol{\theta})|\left[2\left(|\frac{\partial\psi(\boldsymbol{\theta})}{\partial\theta_i}\rangle\langle\frac{\partial\psi(\boldsymbol{\theta})}{\partial\theta_j}|+|\frac{\partial\psi(\boldsymbol{\theta})}{\partial\theta_i}\rangle\langle\psi(\boldsymbol{\theta})|\langle\psi(\boldsymbol{\theta})|\frac{\partial\psi(\boldsymbol{\theta})}{\partial\theta_j}\rangle+\right.\right. \\
&\quad \left.\left.|\psi(\boldsymbol{\theta})\rangle\langle\psi(\boldsymbol{\theta})|\langle\frac{\partial\psi(\boldsymbol{\theta})}{\partial\theta_i}|\frac{\partial\psi(\boldsymbol{\theta})}{\partial\theta_j}\rangle+|\psi(\boldsymbol{\theta})\rangle\langle\frac{\partial\psi(\boldsymbol{\theta})}{\partial\theta_j}|\langle\frac{\partial\psi(\boldsymbol{\theta})}{\partial\theta_i}|\psi(\boldsymbol{\theta})\rangle+h.c.\right)\right]|\psi(\boldsymbol{\theta})\rangle \\
&= 2\left\{2Re[\langle\psi(\boldsymbol{\theta})|\frac{\partial\psi(\boldsymbol{\theta})}{\partial\theta_i}\rangle\langle\frac{\partial\psi(\boldsymbol{\theta})}{\partial\theta_j}|\psi(\boldsymbol{\theta})\rangle]+2\langle\psi(\boldsymbol{\theta})|\frac{\partial\psi(\boldsymbol{\theta})}{\partial\theta_i}\rangle\langle\psi(\boldsymbol{\theta})|\frac{\partial\psi(\boldsymbol{\theta})}{\partial\theta_j}\rangle\right. \\
&\quad \left.+2Re[\langle\frac{\partial\psi(\boldsymbol{\theta})}{\partial\theta_i}|\frac{\partial\psi(\boldsymbol{\theta})}{\partial\theta_j}\rangle]+2\langle\frac{\partial\psi(\boldsymbol{\theta})}{\partial\theta_i}|\psi(\boldsymbol{\theta})\rangle\langle\frac{\partial\psi(\boldsymbol{\theta})}{\partial\theta_j}|\psi(\boldsymbol{\theta})\rangle\right\},
\end{aligned} \tag{S10}$$

where $h.c.$ stands for "Hermitian conjugate".

Denoting $\hat{h}_i=i(\partial\hat{U}^\dagger/\partial\theta_i)\hat{U}$, we have

$$\langle\psi(\boldsymbol{\theta})|\frac{\partial\psi(\boldsymbol{\theta})}{\partial\theta i}\rangle=\langle\psi_0|\hat{U}^\dagger(\boldsymbol{\theta})\frac{\partial\hat{U}(\boldsymbol{\theta})}{\partial\theta i}|\psi_0\rangle=i\langle\hat{h}_i\rangle_0, \tag{S11}$$

$$\langle\frac{\partial\psi(\boldsymbol{\theta})}{\partial\theta_i}|\frac{\partial\psi(\boldsymbol{\theta})}{\partial\theta_j}\rangle=\langle\psi_0|\frac{\partial\hat{U}^\dagger(\boldsymbol{\theta})}{\partial\theta i}\frac{\partial\hat{U}(\boldsymbol{\theta})}{\partial\theta j}|\psi_0\rangle=\langle\psi_0|\frac{\partial\hat{U}^\dagger(\boldsymbol{\theta})}{\partial\theta i}\hat{U}(\boldsymbol{\theta})\hat{U}^\dagger(\boldsymbol{\theta})\frac{\partial\hat{U}(\boldsymbol{\theta})}{\partial\theta j}|\psi_0\rangle=\langle\hat{h}_i\hat{h}_j\rangle_0. \tag{S12}$$

In this way, the QFI matrix is given by

$$I_Q(\boldsymbol{\theta})_{ij}=4[\langle\hat{h}_i\hat{h}_j\rangle_0-\langle\hat{h}_i\rangle_0\langle\hat{h}_j\rangle_0], \tag{S13}$$

provided $[\hat{h}_i,\hat{h}_j]=0$.

We then give an expression for $C_Q(\boldsymbol{\theta},\hat{\Pi}_{\boldsymbol{l}})$, in terms of the initial probe state and the chosen Kraus representation. Note that in the following derivation we have used the properties that $\hat{U}^{(SE)}=\otimes_{i=0}^{d}\hat{U}_i^{(S_iE_i)}$, $\partial\hat{U}^{(SE)}/\partial\theta_i=\partial\hat{U}_i^{(S_iE_i)}/\partial\theta_i\otimes_{k\neq i}\hat{U}_k^{(S_kE_k)}$, $\hat{\Pi}_{l_i}^{(i)}(\theta_i)={}_{E_i}\langle l_i|\hat{U}_i^{(S_iE_i)}(\theta_i)|0\rangle_{E_i}$, $\sum_{l_i}\hat{\Pi}_{l_i}^{(i)\dagger}\hat{\Pi}_{l_i}^{(i)}=I$ and $\sum_{\boldsymbol{l}}|\boldsymbol{l}\rangle\langle\boldsymbol{l}|=I$. For $i\neq j$,

$$\begin{aligned}
C_Q(\boldsymbol{\theta},\hat{\Pi}_{\boldsymbol{l}})_{ij} &= 4\left\{{}_E\langle 0|_S\langle\psi_0|\frac{\partial\hat{U}^{(SE)\dagger}}{\partial\theta_i}\frac{\partial\hat{U}^{(SE)}}{\partial\theta_j}|\psi_0\rangle_S|0\rangle_E-\right. \\
&\quad \left.{}_E\langle 0|_S\langle\psi_0|i\frac{\partial\hat{U}^{(SE)\dagger}}{\partial\theta_i}\hat{U}^{(SE)}|\psi_0\rangle_S|0\rangle_E\,{}_E\langle 0|_S\langle\psi_0|i\frac{\partial\hat{U}^{(SE)\dagger}}{\partial\theta_j}\hat{U}^{(SE)}|\psi_0\rangle_S|0\rangle_E\right\} \\
&= 4\left\{\sum_{\boldsymbol{l}}{}_E\langle 0|_S\langle\psi_0|\frac{\partial\hat{U}^{(SE)\dagger}}{\partial\theta_i}|\boldsymbol{l}\rangle_E\,{}_E\langle\boldsymbol{l}|\frac{\partial\hat{U}^{(SE)}}{\partial\theta_j}|\psi_0\rangle_S|0\rangle_E-\right. \\
&\quad \left.\sum_{\boldsymbol{l}}{}_E\langle 0|_S\langle\psi_0|i\frac{\partial\hat{U}^{(SE)\dagger}}{\partial\theta_i}|\boldsymbol{l}\rangle_E\,{}_E\langle\boldsymbol{l}|\hat{U}^{(SE)}|\psi_0\rangle_S|0\rangle_E\sum_{\boldsymbol{l}}{}_E\langle 0|_S\langle\psi_0|i\frac{\partial\hat{U}^{(SE)\dagger}}{\partial\theta_j}|\boldsymbol{l}\rangle_E\,{}_E\langle\boldsymbol{l}|\hat{U}^{(SE)}|\psi_0\rangle_S|0\rangle_E\right\},
\end{aligned} \tag{S14}$$

note that

$$
\begin{aligned}
{}_E\langle \boldsymbol{l}|\frac{\partial \hat{U}^{(SE)}}{\partial\theta_j}|0\rangle_E &= {}_E\langle \boldsymbol{l}|\frac{\partial \hat{U}_i^{(S_iE_i)}}{\partial\theta_i}\otimes_{k\neq i}\hat{U}_k^{(S_kE_k)}|0\rangle_E \\
&= {}_{E_i}\langle l_i|\frac{\partial \hat{U}_i^{(S_iE_i)}}{\partial\theta_i}|0\rangle_{E_i}\otimes_{k\neq i}{}_{E_k}\langle l_k|\hat{U}_k^{(S_kE_k)}|0\rangle_{E_k} \\
&= \frac{d\hat{\Pi}_{l_i}^{(i)}}{d\theta_i}\otimes_{k\neq i}\hat{\Pi}_{l_k}^{(k)},
\end{aligned}
\tag{S15}
$$

and

$$
\begin{aligned}
&{}_E\langle 0|_S\langle\psi_0|\frac{\partial \hat{U}^{(SE)\dagger}}{\partial\theta_i}|\boldsymbol{l}\rangle_{EE}\langle \boldsymbol{l}|\frac{\partial \hat{U}^{(SE)}}{\partial\theta_j}|\psi_0\rangle_S|0\rangle_E \\
&= {}_S\langle\psi_0|[\frac{d\hat{\Pi}_{l_i}^{(i)\dagger}}{d\theta_i}\otimes_{k\neq i}\hat{\Pi}_{l_k}^{(k)\dagger}][\frac{d\hat{\Pi}_{l_j}^{(j)}}{d\theta_j}\otimes_{k\neq j}\hat{\Pi}_{l_k}^{(k)}]|\psi_0\rangle_S \\
&= {}_S\langle\psi_0|[\frac{d\hat{\Pi}_{l_i}^{(i)\dagger}}{d\theta_i}\hat{\Pi}_{l_j}^{(j)\dagger}\otimes_{k\neq i,j}\hat{\Pi}_{l_k}^{(k)\dagger}][\frac{d\hat{\Pi}_{l_j}^{(j)}}{d\theta_j}\hat{\Pi}_{l_i}^{(i)}\otimes_{k\neq i,j}\hat{\Pi}_{l_k}^{(k)}]|\psi_0\rangle_S \\
&= {}_S\langle\psi_0|\frac{d\hat{\Pi}_{l_i}^{(i)\dagger}}{d\theta_i}\hat{\Pi}_{l_i}^{(i)}\hat{\Pi}_{l_j}^{(j)\dagger}\frac{d\hat{\Pi}_{l_j}^{(j)}}{d\theta_j}\otimes_{k\neq i,j}\hat{\Pi}_{l_k}^{(k)\dagger}\hat{\Pi}_{l_k}^{(k)}|\psi_0\rangle_S,
\end{aligned}
\tag{S16}
$$

so we have

$$
\begin{aligned}
C_Q(\boldsymbol{\theta},\hat{\Pi}_{\boldsymbol{l}})_{ij} &= 4\Bigg\{\sum_{l_il_j}\sum_{\{l_k\}}{}_S\langle\psi_0|\frac{d\hat{\Pi}_{l_i}^{(i)\dagger}}{d\theta_i}\hat{\Pi}_{l_i}^{(i)}\hat{\Pi}_{l_j}^{(j)\dagger}\frac{d\hat{\Pi}_{l_j}^{(j)}}{d\theta_j}\otimes_{k\neq i,j}\hat{\Pi}_{l_k}^{(k)\dagger}\hat{\Pi}_{l_k}^{(k)}|\psi_0\rangle_S- \\
&\quad \sum_{l_i}\sum_{\{l_k\}}{}_S\langle\psi_0|i\frac{d\hat{\Pi}_{l_i}^{(i)\dagger}}{d\theta_i}\hat{\Pi}_{l_i}^{(i)}\otimes_{k\neq i}\hat{\Pi}_{l_k}^{(k)\dagger}\hat{\Pi}_{l_k}^{(k)}|\psi_0\rangle_S\sum_{l_j}\sum_{\{l_k\}}{}_S\langle\psi_0|i\frac{d\hat{\Pi}_{l_j}^{(j)\dagger}}{d\theta_j}\hat{\Pi}_{l_j}^{(j)}\otimes_{k\neq j}\hat{\Pi}_{l_k}^{(k)\dagger}\hat{\Pi}_{l_k}^{(k)}|\psi_0\rangle_S\Bigg\} \\
&= 4\sum_{l_il_j}\Bigg\{{}_S\langle\psi_0|\frac{d\hat{\Pi}_{l_i}^{(i)\dagger}}{d\theta_i}\hat{\Pi}_{l_i}^{(i)}\hat{\Pi}_{l_j}^{(j)\dagger}\frac{d\hat{\Pi}_{l_j}^{(j)}}{d\theta_j}|\psi_0\rangle_S - {}_S\langle\psi_0|i\frac{d\hat{\Pi}_{l_i}^{(i)\dagger}}{d\theta_i}\hat{\Pi}_{l_i}^{(i)}|\psi_0\rangle_{SS}\langle\psi_0|i\frac{d\hat{\Pi}_{l_j}^{(j)\dagger}}{d\theta_j}\hat{\Pi}_{l_j}^{(j)}|\psi_0\rangle_S\Bigg\} \\
&= 4\{\langle\hat{A}^{(i)}\hat{A}^{(j)}\rangle_0-\langle\hat{A}^{(i)}\rangle_0\langle\hat{A}^{(j)}\rangle_0\},
\end{aligned}
\tag{S17}
$$

where

$$
\hat{\Pi}_{l_i}^{(i)} = {}_{E_i}\langle l_i|\hat{U}_i^{(S_iE_i)}|0\rangle_{E_i},
\tag{S18}
$$

$$
\hat{A}^{(i)} = \sum_{l_i} i\frac{d\hat{\Pi}_{l_i}^{(i)\dagger}}{d\theta_i}\hat{\Pi}_{l_i}^{(i)}.
\tag{S19}
$$

For diagonal elements of C_Q, we have

$$
C_Q(\boldsymbol{\theta},\hat{\Pi}_{\boldsymbol{l}})_{ii} = 4\{\langle\hat{B}^{(i)}\rangle_0-\langle\hat{A}^{(i)}\rangle_0^2\},
\tag{S20}
$$

where

$$
\hat{B}^{(i)} = \sum_{l_i}\frac{d\hat{\Pi}_{l_i}^{(i)\dagger}}{d\theta_i}\frac{d\hat{\Pi}_{l_i}^{(i)}}{d\theta_i}.
\tag{S21}
$$

Or we shall write

$$
C_Q(\boldsymbol{\theta},\hat{\Pi}_{\boldsymbol{l}})_{ij} = 4\{\langle\hat{B}^{(ij)}\rangle_0-\langle\hat{A}^{(i)}\rangle_0\langle\hat{A}^{(j)}\rangle_0\},
\tag{S22}
$$

with $\hat{B}^{(ij)} = \hat{B}^{(i)}$ for $i=j$, and $\hat{B}^{ij} = \hat{A}^i\hat{A}^j$ for $i\neq j$.

A CONJECTURE ABOUT THE ATTAINABILITY OF THE QFI BOUND WITH PHOTON LOSS

The initial state is $|\psi_0\rangle = \sum_{k=1}^{D} \alpha_k |N_{k,0}, N_{k,1}, \cdots, N_{k,d}\rangle = \sum_{k=1}^{D} \alpha_k |\boldsymbol{N}_k\rangle$ with the condition that $\sum_{i=0}^{d} N_{k,i} = N$ for any k, where N is the fixed total photon number. We propose that if are α_k all real, then we have $Tr\left[\rho(\boldsymbol{\theta})[L_i, L_j]\right] = 0$ for every L_i and L_j, so that the QFI bound is actually attainable, if the conjecture Eq.(S5) is right. The proof is as follows.

If we write $\rho(\boldsymbol{\theta})$ in its eigenbasis $\rho(\boldsymbol{\theta}) = \sum_n p_n |\psi_n\rangle\langle\psi_n|$, then L_i can be explicitly written as [2]

$$L_i = 2\sum_{mn} \frac{\langle\psi_m|\partial_i\rho(\theta)|\psi_n\rangle}{p_n + p_m} |\psi_m\rangle\langle\psi_m|, \tag{S23}$$

where the sums only include terms with $p_m + p_n \neq 0$. Since $\rho(\boldsymbol{\theta}) = \sum_{\boldsymbol{l}} \hat{\Pi}_{\boldsymbol{l}}(\boldsymbol{\theta})\rho_0\hat{\Pi}^{\dagger}_{\boldsymbol{l}}(\boldsymbol{\theta})$ and $\hat{\Pi}^{(i)}_{l_i} = \sqrt{\frac{(1-\eta_i)^{l_i}}{l_i!}} e^{i\theta_i\hat{n}_i}\eta_i^{\frac{\hat{n}_i}{2}}\hat{a}_i^{\,l_i}$, it can be seen that $\partial_i\rho(\boldsymbol{\theta}) = i\hat{n}_i\rho(\boldsymbol{\theta}) + \rho(\boldsymbol{\theta})(-i\hat{n}_i)$. We then have

$$L_i = 2\sum_{mn} \frac{i(p_n - p_m)}{p_n + p_m} \langle\psi_m|\hat{n}_i|\psi_n\rangle |\psi_m\rangle\langle\psi_m|, \tag{S24}$$

and

$$Tr[\rho(\boldsymbol{\theta})L_iL_j] = 4\sum_{mn} \frac{p_m(p_n - p_m)^2}{(p_n + p_m)^2} \langle\psi_m|\hat{n}_i|\psi_n\rangle\langle\psi_n|\hat{n}_j|\psi_m\rangle. \tag{S25}$$

Suppose $|\varphi\rangle$ is an eigenstate of $\rho(\boldsymbol{\theta})$ with eigenvalue λ, we shall have

$$\lambda|\varphi\rangle = \rho(\boldsymbol{\theta})|\varphi\rangle = \sum_{\boldsymbol{l}} \hat{\Pi}_{\boldsymbol{l}}(\boldsymbol{\theta})\rho_0\hat{\Pi}^{\dagger}_{\boldsymbol{l}}(\boldsymbol{\theta})|\varphi\rangle = \sum_{\boldsymbol{l}} |\varphi_{\boldsymbol{l}}\rangle\langle\varphi_{\boldsymbol{l}}|\varphi\rangle, \tag{S26}$$

with $|\varphi_{\boldsymbol{l}}\rangle = \hat{\Pi}_{\boldsymbol{l}}|\psi_0\rangle$. We see that if $\lambda \neq 0$, $|\phi\rangle$ can be written as a linear combination of $|\varphi_{\boldsymbol{l}}\rangle$.

We shall now give some properties of $|\varphi_{\boldsymbol{l}}\rangle$. First, $\langle\varphi_{\boldsymbol{l}}|\varphi_{\boldsymbol{l}'}\rangle$ is real. To see this, we notice that the form of the Kraus operators are $\hat{\Pi}^{(i)}_{l_i} = \sqrt{\frac{(1-\eta_i)^{l_i}}{l_i!}} e^{i\theta_i\hat{n}_i}\eta_i^{\frac{\hat{n}_i}{2}}\hat{a}_i^{\,l_i}$, since we have assumed that the coefficients of $|\psi_0\rangle$ are real, the only complex number is $e^{i\theta_i\hat{n}_i}$. We focus our attention on node i, and calculate ${}_i\langle n_i'|\hat{\Pi}^{\dagger}_{\boldsymbol{l}'}\hat{\Pi}_{\boldsymbol{l}}|n_i\rangle_i$, we should find that it must be a real number, because after the effect of $\hat{a}_i$, $\hat{\Pi}_{\boldsymbol{l}}|n_i\rangle_i$ and $\hat{\Pi}_{\boldsymbol{l}'}|n_i'\rangle_i$ should be the same fock state, otherwise their inner product must be zero. If they are the same fock state, then the coefficient $e^{i\theta_i n_i}$ will cancel to make zero. In this way, we find that $\langle\varphi_{\boldsymbol{l}}|\varphi_{\boldsymbol{l}'}\rangle$ is real. Following the same proof, we find other properties of $|\varphi_{\boldsymbol{l}}\rangle$: $\langle\varphi_{\boldsymbol{l}}|\hat{n}_i|\varphi_{\boldsymbol{l}'}\rangle$ is real, $\langle\varphi_{\boldsymbol{l}}|\hat{n}_i\hat{n}_j|\varphi_{\boldsymbol{l}'}\rangle$ is real.

Now we focus on the right hand of Eq.(S25). $|\psi_m\rangle$ should be eigenstates with nonzero eigenvalue, otherwise the rhs of Eq.(S25) is zero. On the other hand, $|\psi_n\rangle$ may have nonzero or zero eigenvalues. For those eigenstates with nonzero eigenvalue, we turn to Eq.(S26), we see that it should be a combination of $|\varphi_{\boldsymbol{l}}\rangle$. Further, from the above properties of $|\varphi_{\boldsymbol{l}}\rangle$, we can actually find a set of eigenstates being real combinations of $|\varphi_{\boldsymbol{l}}\rangle$, meaning that $\langle\varphi_{\boldsymbol{l}}|\varphi\rangle$ are all real. Now we see that the contribution of these states to the rhs of Eq.(S25) should be real.

For states with zero eigenvalues, which we denote as $|\psi_n^{zero}\rangle$, also we denote states with nonzero eigenvalues as $|\psi_n^{nonzero}\rangle$, we have

$$\begin{aligned} & 4\sum_{mn} \frac{p_m(p_n - p_m)^2}{(p_n + p_m)^2} \langle\psi_m|\hat{n}_i|\psi_n^{zero}\rangle\langle\psi_n^{zero}|\hat{n}_j|\psi_m\rangle \\ = \;& 4\sum_m p_m\langle\psi_m|\hat{n}_i(\sum_n |\psi_n^{zero}\rangle\langle\psi_n^{zero}|)\hat{n}_j|\psi_m\rangle \\ = \;& 4\sum_m p_m\langle\psi_m|\hat{n}_i(I - \sum_n |\psi_n^{nonzero}\rangle\langle\psi_n^{nonzero}|)\hat{n}_j|\psi_m\rangle, \end{aligned} \tag{S27}$$

we see that it is still a real number. Now we have proven that $Tr\left[\rho(\boldsymbol{\theta})L_iL_j\right]$ is real, such that $Tr\left[\rho(\boldsymbol{\theta})[L_i, L_j]\right] = 0$. In this way, the QFI bound is achievable with those states $|\psi_0\rangle$ with real coefficients α_k if the conjecture Eq.(S15) is right.

TRANSITION OF UNCERTAINTY FOR THE GENERALIZED $N00N$ STATE $|\psi_s\rangle$ WITH PHOTON LOSS

With the same method for calculating C_Q as in the single phase case [7], we have $\hat{A}^{(i)} = a_i\hat{n}_i$, $\hat{B}^{(ij)} = \hat{A}^{(i)}\hat{A}^{(j)}$ for $i \neq j$ and $\hat{B}^{(ii)} = a_i^2\hat{n}_i^2 + b_i\hat{n}_i$, with $a_i = 1-(1+\delta_i)(1-\eta_i)$, $b_i = (1+\delta_i)^2\eta_i(1-\eta_i)$. We investigate the symmetric case that all η_i are the same, such that $\eta_i = \eta$ for any i, and we assume that $\eta < 1$. In order to make the lower bound C_Q of I_Q as tight as possible, we need to optimize all δ_i. To further simplify the calculation, we assume that all δ_i are the same, such that $\delta_i = \delta$ for any i, and now we have only one variable δ to optimize. Take the initial probe to be the generalized $N00N$ state $|\psi_s\rangle$ [8], explicitly written as

$$|\psi_s\rangle = \alpha(|0,N,0,...,N\rangle + |0,0,N,...,0\rangle + \cdots + |0,0,0,...,N\rangle) + \beta|N,0,0,...,0\rangle, \tag{S28}$$

where $\alpha^2 = \frac{1}{d+\sqrt{d}}$ and $d\alpha^2 + \beta^2 = 1$. In this case all the diagonal elements of C_Q are the same, denoted by c_d, and all the off-diagonal elements of C_Q are the same, denoted by c_o. We have

$$\begin{aligned} c_d &= 4\{a^2\langle\Delta\hat{n}_i^2\rangle_0 + b\langle\hat{n}_i\rangle_0\} \\ &= 4\{(1-\sqrt{\frac{1-\eta}{\eta}}\sqrt{b})^2N^2(\alpha^2-\alpha^4) + bN\alpha^2\}, \end{aligned} \tag{S29}$$

and

$$\begin{aligned} c_o = &= 4\{a^2(\langle\hat{n}_i\hat{n}_j\rangle_0 - \langle\hat{n}_i\rangle_0\langle\hat{n}_j\rangle_0)\} \\ &= -4(1-\sqrt{\frac{1-\eta}{\eta}}\sqrt{b})^2N^2\alpha^4, \end{aligned} \tag{S30}$$

where $a = 1-(1+\delta)(1-\eta)$, $b = (1+\delta)^2\eta(1-\eta)$ and i, j are any two different modes chosen from $1, 2, ..., d$. Since δ can be any real number to make $Tr[C_Q^{-1}]$ larger, b is chosen from the whole set of positive real numbers. We can then get the eigenvalues of C_Q, which is $c_d - c_0(d-1$ degnerate) and $c_d + (d-1)c_o$(nondegenerate), such that

$$\begin{aligned} Tr[C_Q^{-1}] &= \frac{d-1}{c_d-c_o} + \frac{1}{c_d+(d-1)c_o} \\ &= \frac{1}{4}\left\{\frac{d-1}{a^2N^2\alpha^2+bN\alpha^2} + \frac{1}{a^2N^2(\alpha^2-d\alpha^4)+bN\alpha^2}\right\} \\ &= \frac{1}{4}\left\{\frac{(d-1)(d+\sqrt{d})}{a^2N^2+bN} + \frac{(d+\sqrt{d})^2}{a^2N^2((d+\sqrt{d})-d)+bN(d+\sqrt{d})}\right\}. \end{aligned} \tag{S31}$$

We consider the asymptotic case that $d \gg 1$, and then the second term of the rhs of Eq. (S31) is infinitesimal compared with the first term such that

$$\begin{aligned} Tr[C_Q^{-1}] &\approx \frac{1}{4}\frac{(d-1)(d+\sqrt{d})}{a^2N^2+bN} \\ &= \frac{1}{4}\frac{(d-1)(d+\sqrt{d})}{(1-\sqrt{\frac{1-\eta}{\eta}}\sqrt{b})^2N^2+bN} \\ &\geq \frac{1}{4}\frac{(d-1)(d+\sqrt{d})}{(1-\frac{\eta'N}{\eta'N+1})^2N^2+\frac{\eta'N^2}{(\eta'N+1)^2}N} \\ &\approx \frac{1}{4}\frac{1}{\frac{1}{(\eta'N+1)^2}(\frac{N}{d})^2+\frac{\eta'N^2}{(\eta'N+1)^2}\frac{N}{d}\frac{1}{d}}, \end{aligned} \tag{S32}$$

where $\eta' = \frac{1-\eta}{\eta}$, and the minimum is obtained for $\sqrt{b} = \frac{\sqrt{\eta'}N}{\eta'N+1}$ or $\delta = \frac{N/\eta}{\frac{1-\eta}{\eta}N+1} - 1$. This result clearly exhibits the transition from the Heisenberg scale with the $O(d)$ advantage to the SQL scale without the $O(d)$ advantage. For $\eta'N \ll 1$ or $\frac{1-\eta}{\eta} \ll \frac{1}{N}$, we have $Tr[C_Q^{-1}] = \frac{1}{4}\frac{1}{(N/d)^2}$, whereas for $\eta'N \gg 1$ or $N \gg \frac{\eta}{1-\eta}$, we have $Tr[C_Q^{-1}] = \frac{1-\eta}{4\eta}\frac{1}{N/d}d$.

---

* Electronic address: hfan@iphy.ac.cn

[1] C. Helstrom, *Quantum Detection and Estimation Theory, Mathematics in Science and engineering* (Academic Press, Massachusetts, 1976).
[2] M. G. A. Paris, Int. J. Quant. Infor. **07**, 125 (2009).
[3] M. A. Nielsen and I. L. Chuang, *Quantum Computation and Quantum Information* (Cambridge University, Cambridge, England, 2001).
[4] R. D. Gill and S. Massar, Phys. Rev. A **61**, 042312 (2000).
[5] M. G. G. Cyril Vaneph and T. Tufarelli, Quantum estimation of a two-phase spin rotation, *Quantum Measurements and Quantum Metrology , 1*, 12-20 (2013).
[6] R. A. Horn, C. R. Johnson, *Matrix Analysis* (Cambridge University Press, 1990).
[7] B. Escher, R. de Matos Filho, and L. Davidovich, Nature Physics **7**, 406 (2011).
[8] P. C. Humphreys, M. Barbieri, A. Datta, and I. A. Walmsley, Phys. Rev. Lett. **111**, 070403 (2013).
[9] Richard D. Gill, Madalin Guta, eprint arXiv:1112:2078.
